# Book Review

**DOI:** 10.1080/10543400802536305

**Published:** 2009-01-07

**Authors:** Steve Simon

**Affiliations:** P. Mean Consulting, 14814 Granada Court, Leawood, KS 66224

I suspect that Dr. Senn is a hero to many readers of this journal. He has been a fervent advocate of the quality of work done by pharmaceutical statisticians in a day and age where many dismiss any research touched by commercial interests (Senn, 2005, 2006). He also argues loud and forcefully for patients when their lives are trampled by unethical demands of the research enterprise (Senn, 1997, 2003a). He has written the definitive work on crossover trials (Senn, 2002), and a wonderfully readable account of statistics in medicine for the lay public (Senn, 2003b). He is the source of untold delightful quotes. My favorite is his definition of medical statistician: “one who will not accept that Columbus discovered America … because he said he was looking for India in the trial plan.” Finally, he is an all-around smart guy who has moved the statistics discipline forward in many different areas. Dr. Senn is one of the few people whose books you would be well advised to buy based on his reputation alone.

The second edition of *Statistical Issues in Drug Development* updates a book originally published in 1997 and it maintains much of the same organization. Dr. Senn has provided word counts for each chapter in the first and second edition. The average chapter has increased by about 2,000 words, with most of the increases occurring in the second half of the book.

The first five chapters of *Statistical Issues in Drug Development* offer a very general perspective, including a nice historical overview. The discussion of the proper role of a statistician in a pharmaceutical company will help others to appreciate the depth and breadth of our contributions, but Dr. Senn also holds us to a very high standard. I was humbled, for example, by a discussion on page 58 of how a statistician familiar with pulmonary function testing might be well-positioned to discuss the implications on design and sample size when peak expiratory flow is substituted for forced expiratory volume. I've worked with such measures for more than two decades, but I doubt that I have sufficient medical appreciation of these tests to discuss this topic at the level suggested here.

The remaining 20 chapters of the book cover specific topics such as baseline adjustments, subgroup analysis, multiplicity, intention-to-treat analysis, multicenter trials, equivalence studies, meta-analysis, cross-over trials, *n*-of-1 trials, sequential trials, dose-finding, pharmacokinetics/dynamics, pharmacoepidemiology, and pharmacoeconomics. A new chapter in the second edition covers pharmacogenetics.

Discussion of the controversy between frequentists and Bayesians is distributed through many different chapters of this book. Dr. Senn has a dispassionate discussion of these and other issues, which is a rare and welcome change. When Dr. Senn does not like a commonly used approach, he finds a way to disagree without being disagreeable.

Dr. Senn leaves almost no stone uncovered. There is no discussion of medical devices, but this cannot be considered an omission in a book with the words “drug development” in the title. I do worry about Dr. Senn's failure to mention any special issues related to pediatrics. Regulators are increasingly mandating special testing to allow appropriate dosing information for children (Hampton, 2004), and these studies raise special challenges for everyone, including the statisticians. In general, the book provides an excellent coverage to such a broad and diverse area.

One needs to be careful in describing the audience for a book like this. A practicing statistician will find that this book does not cover any particular topic in the level of detail that such a person needs. You won't be able to properly design and analyze a group sequential trial, for example, after reading the chapter on this topic. What this book provides is a gentle introduction to an area, an outline of the major controversies in that area, and references for anyone who wants to dig further. If you are moving into an area of drug development that is new to you, (say, dose-finding) then this book can jump start your transition, but this won't be the book that you constantly reach for as you hone your skills.

Statisticians with limited experience in drug development will greatly benefit from seeing the careful layout of controversies that are unique to this arena. I especially loved the description of the controversies associated with intention-to-treat analysis and random effects in a multicenter trial.

Another possible audience is researchers who want to develop a greater degree of sophistication in their work by better understanding the statistical issues associated with the design and analysis of drug development studies. You may want this book, just to help answer questions from some of your more sophisticated clients. To help some of the math-phobic clients, Dr. Senn segregates most formulas to an appendix at the end of the chapter.

It won't help, though, for your unsophisticated clients. This is not a *Statistics for Idiots* book. Even with the mathematics removed, the intellectual caliber required to appreciate this book is still substantial.

If you own the first edition of the book should you buy the second? An entirely new chapter on pharmacogenomics is nice, but not worth the extra money all by itself. A better justification is that the controversies discussed in the first edition have in most cases heated up rather than settled down. You can't pretend you're on top of these issues with a book that is a decade old. The bibliographies at the end of each chapter reflect this. Of the 953 total references, 634 are new to this edition and the three quartiles of publication year for these new references are 1995, 2000, and 2004.

Writing a review of this book reminds me of the Will Rogers quote “We can't all be heroes, because somebody has to sit on the curb and clap as they go by.” This book is an outstanding effort from a statistician of heroic proportions. Someone like me is only capable of sitting on the curb and applauding wildly.
